# Assessment of ADC Higher Order Structure Through 2D NMR Analysis

**DOI:** 10.3390/molecules30224490

**Published:** 2025-11-20

**Authors:** Emily M. Grasso, Angela N. Marquard, Zachary Sparta, David Fry, Nareshkumar Jain

**Affiliations:** 1 Platform Innovation, NJ Bio, Inc., Princeton, NJ 08540, USAdavid.fry@njbio.com (D.F.); 2 Bioconjugation Research and Development, NJ Bio, Inc., Princeton, NJ 08540, USA; 3 Global Operations, NJ Bio, Inc., Princeton, NJ 08540, USA

**Keywords:** biomolecular NMR, antibody-drug conjugates, trastuzumab, MMAE, deruxtecan, drug-antibody ratio

## Abstract

Antibody–drug conjugates (ADCs) represent a growing class of important chemotherapeutic molecules. Our understanding of the physical properties of the antibody, linker, and payload is still quite limited, but a better understanding may lead to superior ADCs. Biomolecular NMR has shown promise in the characterization of antibody higher-order structure, suggesting that the same should be true for ADCs. We applied 2D NMR techniques to trastuzumab alone and to trastuzumab conjugated to MMAE and DXd linker-payloads at drug-antibody ratios (DAR) 2, 4, and 8 to assess the effects of drug conjugation on antibody higher-order structure. Trastuzumab alone generated high-quality NMR spectra under a variety of temperatures and concentrations. Spectra of low DAR species were remarkably similar both to each other and to the free antibody, except for notable new peaks in the spectra from the linker-payloads. Increasing DAR resulted in the disappearance of many well-dispersed peaks; at the highest DAR, both T-MMAE and T-DXd showed a global broadening of signals, although this effect was more extreme in T-MMAE. These spectra demonstrate the promise of biomolecular NMR to provide a direct window into the solution behavior of ADCs.

## 1. Introduction

Antibody–drug conjugates (ADCs) have become an increasingly important therapeutic strategy targeting various cancers over the last 25 years, though the idea has existed for much longer [1,2]. An ADC is comprised of an antibody attached via a linking moiety to a cytotoxic payload, allowing the release of the payload into cells targeted by the antibody [1,3,4,5,6]. While several ADCs have achieved FDA approval, there are many more that have failed in the clinic due to a variety of issues [7,8,9]. Our understanding of the fundamental principles of ADC development, including the physical properties of the antibody, linker, and payload, is still quite limited [4,5,10,11,12]. It is well established that the relationship between the drug-antibody-ratio (DAR) and in vivo activity is nonlinear; that is, adding more drugs onto the ADC does not necessarily lead to greater clinical efficacy [12,13]. Broadening the analytical tools by which we characterize the solution behavior of ADCs is thus critical to improving our understanding of these molecules and producing more efficacious therapies.

Biomolecular NMR has shown promise in the characterization of biologics and assessment of biosimilars, particularly with respect to characterizing the higher-order structural features of these molecules [14,15,16,17,18,19,20,21,22,23,24]. Advances in method development and spectrometer capabilities have facilitated the collection of NMR spectra on full-length antibodies, as well as isolated Fc and Fab regions [15,16,25,26,27]. These techniques have been particularly effective in monitoring for changes due to degradation [15,24,27]. Due to the prohibitive cost of isotopically labeling full-length antibodies, many of these NMR experiments rely on the natural abundance (1% for ^13^C) of methyls within the antibody and, as such, are relatively insensitive. However, local regions within mAbs are sufficiently dynamic to allow for the generation of “fingerprint” methyl spectra that report on the higher-order structure of these extremely large molecules using specifically optimized methyl-selective experiments. Application of similar NMR strategies to ADCs should give us a direct window into the solution behavior of these biomolecules to facilitate the development of better ADCs.

We set out to characterize ADCs using NMR spectroscopy. We turned to trastuzumab, a well characterized, robust antibody with therapeutic applications both alone (Herceptin^®^) and as an ADC (ado-trastuzumab emantasine, Kadcyla^®^, and fam-trastuzumab-deruxtecan-nxki, Enhertu^®^). NMR studies have been performed on both full-length trastuzumab and the isolated Fab and Fc regions, indicating that this system is suitable for detailed analysis and perturbation [15,16,25,26,28]. Both MMAE and DXd are extensively used payloads and have had their activities investigated in vitro and in vivo at different DAR values [7,13,29,30,31,32,33,34]. We collected NMR spectra of both free trastuzumab and ADC versions with either MMAE (T-MMAE) or DXd (T-DXd) as the payload at different DAR values to understand the effect of increasing DAR on the quality of the NMR spectra. These are, to our knowledge, the first published NMR spectra of ADCs, and they highlight the potential of biomolecular NMR as a tool to characterize the solution properties of biologics.

## 2. Results

### 2.1. Trastuzumab Generates Consistent NMR Spectra Under Various Conditions

**Figure 1:**
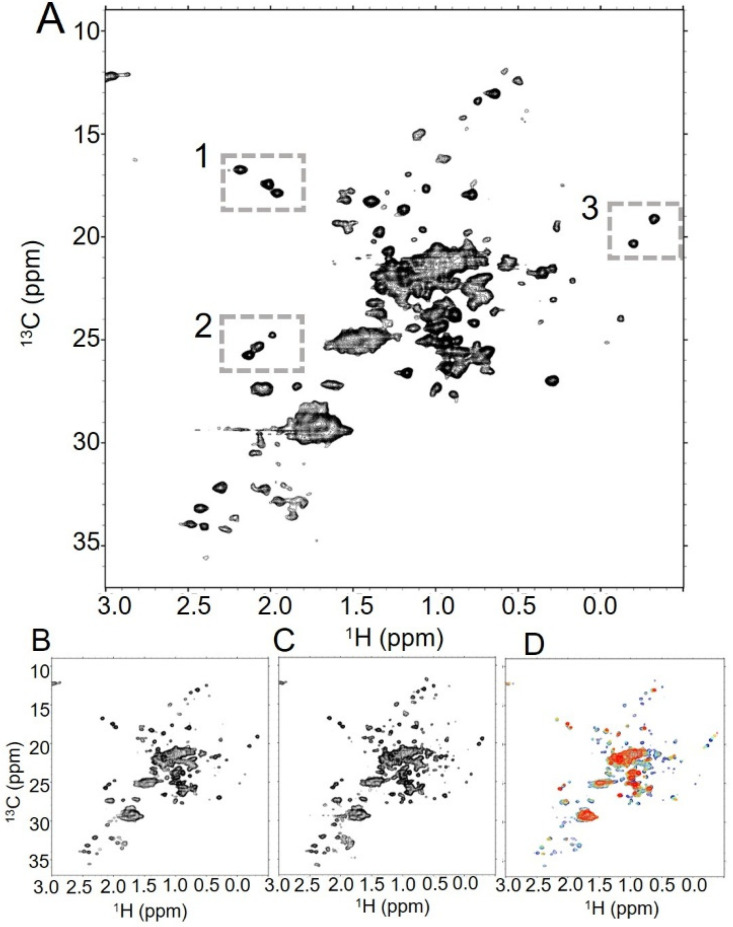
Trastuzumab generates high-quality NMR spectra under various conditions. (**A**) In the {^1^H-^13^C}HOS-XLAFHMQC spectrum of trastuzumab at 37 °C, spectroscopic signatures for trastuzumab are clearly visible, such as the three methionines from Fc (box 1), the glycan N-acetyl groups (box 2), and the upfield crosspeaks that may be attributable to Fab valines (box 3). The number of well-dispersed peaks increases with (**B**) an increased number of scans (128 scans vs. 32 scans in (**A**)), and (**C**) an elevated temperature (50 °C vs. 37 °C in (**A**)). (**D**) Overlaid spectra at 50 °C (blue), 40 °C (aquamarine), 30 °C (gold), and 20 °C (red) reveal a global decrease in signal as temperature lowers, though several peaks in the spectrum persist even at the lowest temperature.

We first collected data on trastuzumab under a variety of conditions to assess both the replicability of previously published data in our systems and establish a range of conditions for future data analysis. At high concentration (29 mg/mL), we found that a spectrum with many dispersed peaks could be collected at 37 °C with 32 scans in slightly over one hour (Figure 1A). Longer data acquisition with more scans led to improved signal-to-noise, but was not essential for visual assessment of spectral quality (Figure 1B). These conditions and spectra are consistent with previously published data for full-length trastuzumab as well as the isolated Fc and Fab [15,25,26]. We next explored the effects of temperature on the spectra to establish optimal conditions for subsequent data collection. As has previously been reported, increasing the temperature of samples from 37 °C to 50 °C increases the number of identifiable peaks with fewer scans without inducing chemical shift changes (Figure 1C, Supplementary Figure S1). Although higher temperatures consistently produce the best spectra, we found that residual signals from specific resonances, including well-dispersed methionine and isoleucine methyls, persist even at temperatures as low as 20 °C (Figure 1D, Supplementary Figure S2). This suggests that these resonances likely exist within highly dynamic regions of the antibody, such that the local dynamics within those regions result in sharp peaks even with the globally slowed tumbling of the antibody at lower temperatures. While spectra containing many dispersed peaks well above the noise level can be acquired in as little as an hour for concentrated trastuzumab, data collected with the same parameters are less robust for lower concentration samples. At concentrations as low as 10 mg/mL, many well-dispersed peaks are visible in spectra with increased experimental time (Supplementary Figure S3). However, further reducing the concentration to 5 mg/mL or 2.5 mg/mL results in the disappearance of many peaks, even with an eight-fold increase in experimental time. We further note the relative stability of trastuzumab spectra over long periods of time. Short spectra collected over the course of one day show minor intensity changes in peaks close to the noise level, but strong signals are unperturbed over this time frame (Supplementary Figure S4A–C). Spectra collected on the same sample after over four months of long periods of data collection and prolonged storage again show extremely minimal changes (Supplementary Figure S4D,E). Together, these data indicate that trastuzumab generates consistent NMR spectra across a wide range of concentrations and temperatures.

### 2.2. T-MMAE NMR Spectra Indicate That the Antibody Structure Is Largely Unchanged by Attachment of the Payload

**Figure 2:**
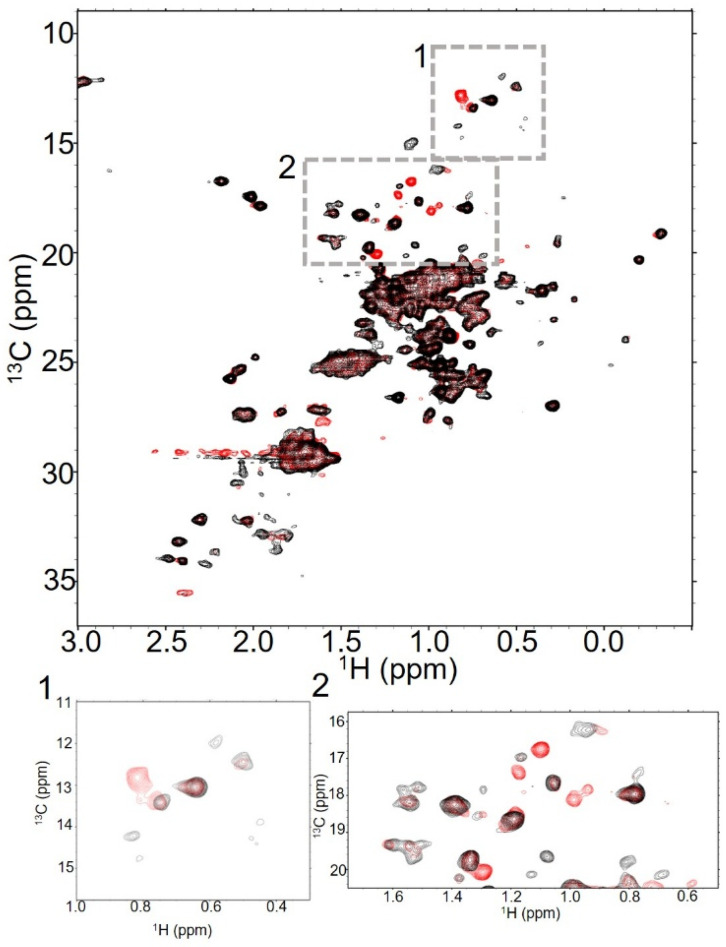
T-MMAE spectra show peaks for both the antibody and payload, but look remarkably like the antibody itself. Overlaid 32 scan NMR spectra of trastuzumab (black) and T-MMAE DAR2.3 (red) show significant similarities in spectra collected at 37 °C. In addition to the significant number of peaks that are in both spectra, there are new peaks in the spectrum of T-MMAE, suggesting that the methyls from the linker-payload are appearing in the spectrum (boxes 1 and 2).

We next collected NMR spectra of T-MMAE, a well established ADC with known in vivo and in vitro behavior. MMAE-based payloads have a long history within the ADC field and are normally conjugated to DAR between 3 and 4 [7]. Given the remarkably stable spectra of the isolated antibody, we initially approached data collection of this ADC and all others using longer experimental times, elevated temperatures, and parameters that worked well for trastuzumab alone. However, we noted that under these conditions, we saw relatively rapid changes in the spectra of the ADC samples, even with low DAR samples. As a result, we prioritized rapid data collection with high-concentration samples at consistent temperatures to ensure the quality of collected data. While the highest temperature for data collection, 50 °C, produced spectra containing the highest number of well-resolved peaks for unconjugated trastuzumab, we chose a more moderate temperature of 37 °C for data collection on ADC samples, as it provided the best balance of generating spectra containing numerous well-resolved peaks and facilitating sample longevity. We collected a series of short and longer spectra on a high-concentration T-MMAE DAR2.3 sample over the course of a day at 37 °C (Figure 2). As with trastuzumab alone, T-MMAE DAR2.3 generates relatively high-quality fingerprint spectra in a short period of time that are improved with longer data acquisition. New, intense peaks appear in the spectrum of T-MMAE DAR2.3 that are likely from the linker-payload (Figure 2, boxed insets). The mc-vc-PAB-MMAE linker-payload should contain 14 methyls, of which 10 might reasonably be expected to resonate within the chemical shift range of these selective experiments. There are at least six new peaks in the spectrum of T-MMAE DAR2.3, some of which may represent more than one methyl. The peaks from the mAb itself remain largely in the same positions, as is evident from the number of overlapping peaks in Figure 2. These data suggest that the antibody structure is largely conserved upon conjugation to the drug and remains symmetric under these conditions. The payload peaks are approximately as intense as the antibody peaks, suggesting that the payload is tumbling at the same rate as the antibody itself. Over the course of one day at 37 °C, minor intensity changes appear within the spectra for relatively intense peaks that were well above the noise in trastuzumab, indicating that this sample was less stable than the antibody alone (Supplementary Figure S5A–C). These changes seem to be localized to specific peaks within the spectrum, indicating that the linker-payload has differential effects on stability within different regions of the antibody. For example, inspection of the isoleucine region of the spectrum above 15 ppm in ^13^C and between 0.5 and 1 ppm in ^1^H reveals four dispersed peaks at the beginning of data collection. After a day, three of those peaks are relatively unchanged, while one is notably attenuated in intensity. At DAR2.3, these changes are relatively minor.

### 2.3. T-MMAE NMR Spectra Show Hallmarks of Aggregation and Instability with Increased DAR

**Figure 3:**
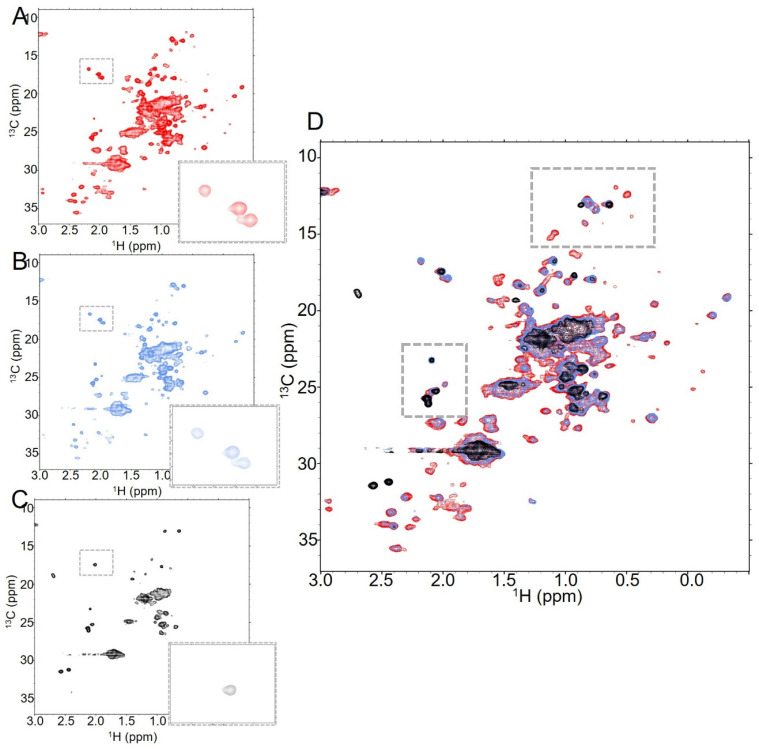
Increasing the DAR of T-MMAE results in a substantial global reduction in signal as well as loss of specific peaks. NMR spectra of T-MMAE collected with 128 scans at (**A**) DAR2.3 (red), (**B**) DAR4.3 (blue) and (**C**) DAR7.9 (black), all collected at 37 °C. The number of dispersed peaks drops significantly as DAR increases, as does the signal from the overlapped peak mass at the center of the spectrum. This is further evident when all three spectra are overlaid, as in (**D**).

We then collected NMR spectra of trastuzumab-MMAE at increasing DAR values to assess how the solution behavior of this ADC, as seen in these NMR experiments, changes with DAR (Figure 3). Increasing from DAR2.3 to DAR4.3 results in a noticeable reduction in signal throughout the spectra, particularly in the overlapped set of signals in the center of the methyl region (Figure 3A,B). A few low-intensity, well-dispersed peaks broaden substantially and cannot be resolved even with longer data collection. However, what peaks are in the spectrum largely remain in the same location, again indicating that the conformational ensemble of the mAb itself is conserved between the DAR2.3 and DAR4.3 species. As with the DAR2.3 sample, spectral changes appear over a day of data collection (Supplementary Figure S5D–F). New peaks grow into the spectra and the relative intensities of specific peaks change. This is particularly evident in the upfield Fab methyls below 0 ppm in ^1^H (Supplementary Figure S5F). At the beginning of data collection, both peaks are visible, but only one remains visible at the end of approximately one day of data collection. In contrast, both of these peaks were visible in DAR2.3 T-MMAE (Supplementary Figure S5C) and trastuzumab (Supplementary Figure S4) over the same time frame.

While the changes in spectral quality between DAR2.3 and DAR4.3 are relatively modest, increasing to DAR7.9 results in a much more dramatic reduction in signal from the protein despite similar concentrations and sample conditions (Figure 3C). This is particularly evident in the concentrated central signal in the spectrum from overlapping methyls, which is substantially broader in the DAR7.9 sample than in the lower DAR samples (Figure 3D). Many of the dispersed peaks in the spectrum broaden into the noise and those that remain have substantially lower intensity. For example, the intensities of the methionine signals visible in the boxed region of the spectra show a strong dependence on DAR. Three methionine methyls are visible in the spectra of trastuzumab and T-MMAE at DAR2.3 and 4.3 at the given contour level (Figure 3A,B, boxed region). In contrast, only one methionine methyl is present in the spectrum of T-MMAE DAR7.9 at the same contour level (Figure 3C, boxed region); while one additional methyl can be seen by lowering the contour levels closer to the noise, the third methyl cannot be seen in the spectrum, regardless of contour level. Together, these data suggest that the local dynamic changes that induce broadening of specific peaks in the DAR2.3 and DAR4.3 spectra are exacerbated in the DAR7.9 sample and are likely compounded by a global dynamic change that causes an overall reduction in signal. Importantly, many of the new peaks that are likely attributable to linker-payload methyls are no longer visible in the DAR7.9 spectra. This indicates that the payload is part of the globally slowed dynamic processes. Additionally, there was a dramatic decrease in the stability of the DAR7.9 sample relative to the lower DAR samples. While spectra of lower DAR samples collected overnight showed relatively modest intensity changes throughout the spectra, noticeable new peaks began to appear and original peaks began to broaden in the T-MMAE DAR7.9 spectra within a few hours of data collection, indicating that new conformations are being populated in the DAR7.9 sample over time (Supplementary Figure S6).

To further investigate the DAR-specific features of our NMR spectra, we compared various properties of the NMR samples from before and after data collection using more traditional metrics, including activity via IC_50_ assays, aggregation via SEC and DAR via HIC, RPLC, and LC-MS (Supplementary Tables S1 and S2, Supplementary Figure S7). In cytotoxicity data collected with pre-NMR samples, all ADCs showed IC_50_ values consistent with expectations, with cell type dependence, suggesting these in vitro data are decoupled from the structural changes indicated by NMR spectra [35,36]. Though the DAR4.3 sample showed a slight increase in aggregation after data collection, only the DAR7.9 sample showed considerable aggregation by SEC, dropping to only >71% monomeric. This is consistent with previous results indicating that high-DAR T-MMAE is prone to aggregation and suggests that the rapid changes that were evident in T-MMAE DAR7.9 spectra are likely due to aggregation within the sample over time [29,30]. However, even initial spectra of T-MMAE DAR7.9 showed decreased dispersion of signals and local changes in peak intensity that suggest poor solution behavior of this ADC relative to lower DAR samples that likely precedes aggregation within the sample. Re-analysis of the DAR for each of the samples post-data collection confirmed that the DAR was unchanged, however, confirming that the spectroscopic changes between samples were due to aggregation and changes in local flexibility rather than the loss of payload.

### 2.4. T-DXd and T-MMAE NMR Spectra Are Remarkably Similar at Low DAR

**Figure 4:**
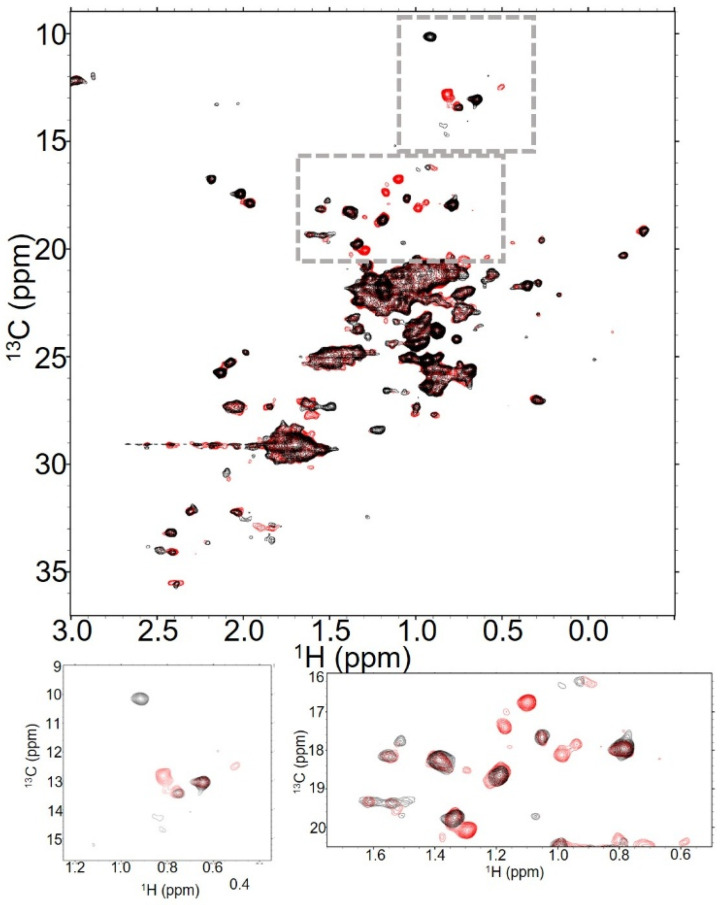
T-DXd and T-MMAE spectra are similar at low DAR. Overlaid 32 scan NMR spectra of T-MMAE at DAR2.3 (red) and T-DXd at DAR2.3 (black) show remarkable similarity in spectra collected at 37 °C. The most significant differences between the spectra are apparent in the signals apparently arising from the payload—while T-MMAE has several new signals, consistent with the presence of many methyls within the linker-payload, the payload produces one substantial peak in T-DXd, consistent with the limited methyl content of the DXd linker-payload.

We next assessed the generalizability of these trends by extending our analysis to T-DXd. Like T-MMAE, this ADC is well characterized by various solution measurements. DXd contains fewer methyls than MMAE, meaning the contribution from the payload itself to the ADC spectra is more straightforward, as is evident from overlaid spectra of T-MMAE and T-DXd DAR2 species (Figure 4). One prominent peak near 1 ppm in ^1^H and 10 ppm in ^13^C seems to come from the DXd payload itself, in contrast to the many dispersed peaks that are likely from the MMAE linker-payload (Figure 4, boxed insets). Visual comparison of the T-DXd and T-MMAE DAR2 spectra reveals many similarities between the two ADCs. In both spectra, the majority of the peaks in the spectra are from the antibody itself. Characteristic dispersed peaks corresponding to methionine and valine side chains are present, as are the peaks corresponding to glycan methyls. There is substantial intensity from overlapped methyl signals near the center of the spectrum as well. The main differences between the spectra arise from the payload peaks, as described above. These data suggest that at low DAR, the identity of the conjugated linker-payload has minimal effects on the local structural features of trastuzumab itself.

### 2.5. T-DXd NMR Spectra Show Fewer Characteristics of Aggregation at Higher DAR

**Figure 5:**
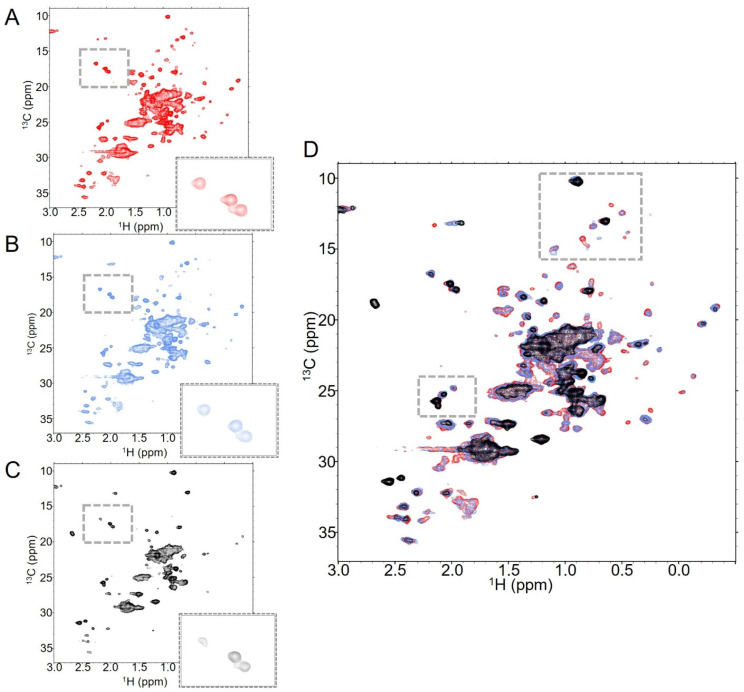
T-DXd retains antibody signals even at high DAR. NMR spectra of T-DXd at (**A**) DAR2.3 (red), (**B**) DAR4.2 (blue) and (**C**) DAR8 (black) show both global and residue-specific changes in 128 scan experiments collected at 37 °C. This is emphasized when all three spectra are overlaid, as in (**D**).

We next collected data with T-DXd at higher DAR values, including DAR4.2 and DAR8 (Figure 5). Comparison of T-DXd DAR2.3 and DAR4.2 spectra reveals many similarities to the T-MMAE low-DAR species. As with T-MMAE, several lower-intensity well-dispersed peaks broaden with elevated DAR (Figure 5A,B). The peaks that remain in the spectra are in the same location. These data indicate that the conformational ensemble adopted by trastuzumab in this ADC is preserved when conjugated to the linker-payload at low DAR.

Increasing the DAR further to DAR8 had a notable impact on the number of identifiable dispersed peaks in the spectrum, with many peaks visible in low-DAR spectra broadened into the noise in DAR8 spectra (Figure 5C). However, there are some notable features within the T-DXd DAR8 spectra that indicate that the global dynamic processes impacting T-MMAE DAR7.9 are likely not as significant in T-DXd DAR8. Of the three Fc methionines that are visible in the spectra of trastuzumab alone, all are still evident in the T-DXd spectra at DAR8, albeit at reduced intensity (Figure 5C inset). The Fab methyl peaks that are below 0 ppm in ^1^H are still present and the overlapped methyl signal at the center of the spectrum is not as substantially broadened as in T-MMAE DAR7.9 (Figure 5D). All of these data indicate that the global dynamics of T-DXd DAR8 are not as dramatically impacted by the linker-payload as T-MMAE at similar DAR. Interestingly, the new methyl signal in T-DXd spectra that is likely from the payload itself does not reduce in intensity at high DAR as it does with T-MMAE. Rather, this peak increases in intensity with increasing DAR, suggesting that the payload is freely rotating in solution independent of the dynamic properties of the antibody itself.

We further noted the apparent stability of T-DXd relative to T-MMAE. While minor spectroscopic changes are apparent in all three datasets at different DAR after a day of data collection at 37 °C, there are no peaks that seem to experience substantial chemical shift changes as in T-MMAE DAR7.9 (Supplementary Figure S8). This leads us to conclude that T-DXd is considerably more stable under the presented conditions than T-MMAE. This interpretation is borne out by SEC analysis, in which all samples remain >96% monomeric before and after data collection, and DAR analysis, which shows no substantial alterations in DAR by various techniques after data collection (Supplementary Table S1, Supplementary Figure S9). Similarly, cytotoxicity assays showed that T-DXd species were functional and IC_50_ values fell into expected ranges (Supplementary Table S2) [32,37].

## 3. Discussion

We describe here the collection and interpretation of NMR spectra of trastuzumab alone, along with trastuzumab conjugated to both MMAE and DXd at different DAR values. These are the first, to our knowledge, published NMR spectra of ADCs. We found that trastuzumab alone generates high-quality NMR spectra under a variety of concentrations and temperatures. Under similar conditions, the dispersion and relative intensities of peaks change dramatically in ADCs. Many of these changes correlate with DAR; as DAR increases, so too does the broadening of peaks within the ADC spectra. The ADC spectra contain a combination of peaks from both the antibody itself and new peaks that are likely from the linker-payloads. The peaks from the antibody do not seem to undergo chemical shift perturbations for the most part, suggesting that the notable symmetry within the antibody is retained even after conjugation.

NMR spectroscopy is, of course, an ensemble measurement, meaning that the spectra of each of these ADCs represent the ensemble of species in each sample. While partial reduction of the antibody to generate the ADCs controls for DAR to an extent, the reported DAR is an average of the species present in the sample, meaning that many possible combinations of DAR are present within the sample that average out to the reported DAR. The trends that we see by NMR are thus population averages of the changes happening throughout these fundamentally heterogeneous samples. Despite the complexity of these samples, however, there are clear changes in specific peaks within these spectra at different DAR values. We noted several well-dispersed peaks with changes in relative intensity at different DAR values, along with global changes in signal from more overlapped peaks. With the recent progress in the assignment of trastuzumab, we will have the opportunity to understand at a deeper level the site-specific changes that occur to antibodies upon conjugation to linker-payloads [25,26]. Chemometric and other similar analyses of ADC spectra may facilitate more robust interpretations of changes with time and payload identity [15,19,38].

Despite these limitations, NMR spectroscopy gives us a window into the solution behavior of these ADCs on a residue-specific level. There are clear visual differences between the spectra presented here, particularly when comparing the spectra collected at high DAR to those at low DAR. It is readily apparent that there are simply fewer peaks within the T-MMAE DAR7.9 sample relative to the other spectra collected. Conjugation of this antibody to this linker-payload at high DAR is well understood to induce instability or aggregation within the ADC that likely accounts for the discrepancy between in vitro and in vivo assessments of ADC function [13]. The conditions chosen for data collection, including high concentration and a 20 mM histidine buffer, which is commonly used in formulation, may have impacted sample stability as well. However, it is notable that under these relatively stressed conditions, we were able to identify fundamental stability differences between the different ADC samples. While aggregation does appear within the T-MMAE DAR7.9 sample after a day spent at 37 °C, spectra collected immediately after sample preparation show broadening, indicating that this ADC is poorly behaved in solution even outside of the time-dependent aggregation. Even T-DXd, which exists as a DAR8 ADC therapeutic, shows substantial spectroscopic changes by NMR with increasing DAR despite all indications of sample quality through traditional ADC assessment, including SEC, HIC, RPLC, LC-MS and cell-based assays. Upon drug conjugation, these ADC spectra simply look less like the antibodies themselves. Application of NMR spectroscopy to study antibodies is gaining traction. The use of similar ideas in the field of ADCs to assess the higher-order structure in solution may pave the way for the development of better ADCs.

## 4. Materials and Methods

### 4.1. Conjugation of T-MMAE and T-DXd

150 mg trastuzumab (5.6 mg/mL in 1× PBS + 1 mM EDTA; commercially sourced, Supplementary Figure S10) was reduced with either 1.4, 2.5, or 10 Eq TCEP (10 mM solution in water) and incubated at 37 °C for ~1.5 h. Each reduction was split into two 75 mg conjugations with either 3.5, 5.5, or 12 Eq mc-vc-PAB-MMAE or Deruxtecan (10 mM solution in DMA) to target DAR 2, 4, or 8, and additional DMA was added to bring the final composition to 10% DMA. The reaction was incubated at room temperature for ~3 h then stored overnight at 4 °C. Conjugates were purified by dialysis (10 K MWCO) into 20 mM Histidine, pH 5.5, concentrated with a 30 K MWCO Vivaspin centrifugal concentrator (Cytiva, Marlborough, MA, USA), and filtered through a 0.22 µm sterile filter.

### 4.2. Characterization of DAR and Monomer

LC-MS analysis of free linker-payloads before conjugation was performed on a Waters Acquity UPLC system connected to a Waters (Milford, MA, USA) Synapt QToF mass spectrometer using a Waters Acquity UPLC CSH C18 column (1.7 µm, 2.1 × 50 mm) in a 5–95% gradient of Acetonitrile/H_2_O containing 0.1% formic acid (Supplementary Figures S11 and S12). Reverse-Phase HPLC and LC-MS analysis of conjugation reactions and products were performed on a Waters Acquity UPLC system connected to a Waters Xevo G2-XS QToF mass spectrometer using an Agilent (Santa Clara, CA, USA) PLRP-S column (1000 Å, 8 µm, 2.1 × 50 mm) in a 25–45% gradient of Acetonitrile/H_2_O containing 0.1% formic acid. Briefly, 25 µg conjugate was deglycosylated with PNGase F (New England Biolabs, Ipswich, MA, USA, P0710S), reduced with DTT or TCEP, and ~1 µg was injected for analysis (Supplementary Figures S13 and S14). DAR was calculated using Equation (1).

(1) $$DAR=2*\;\left(\frac{\sum \left(n*L_{n}\right)}{\sum (L_{n})}+\frac{\sum \left(n*H_{n}\right)}{\sum (H_{n})}\right)$$

Equation (1). DAR by LC-MS/RP-LC calculation in which n refers to the number of payloads, L_n_ refers to the light chain signal with respect to the number of attached payloads, and H_n_ refers to the heavy chain signal with respect to the number of attached payloads.

Hydrophobic-interaction chromatography (HIC) for analysis was performed on a Waters Acquity UPLC using a Tosoh (Griesheim, Germany) TSKgel Butyl-NPR column (2.5 µm, 4.6 mm ID × 10 cm) with a 0–100% gradient. Mobile phase A: 50 mM Na_2_HPO_4_ + 2 M (NH_4_)_2_SO_4_, pH 7.5. Mobile phase B: 50 mM Na_2_HPO_4_ + 20% isopropanol, pH 9.5. ~40 µg conjugate was injected for analysis. DAR was calculated using Equation (2).

(2) $$DAR=\left(\frac{{\sum n*D}_{n}}{\sum D_{n}}\right)$$

Equation (2). DAR by HIC calculation, in which n refers to the number of payloads and D_n_ refers to the signal from the antibody with respect to the number of attached payloads.

Size-exclusion chromatography (SEC) for analysis was performed on an Agilent 1260 Infinity II HPLC system using a Tosoh TSKgel QC-PAK GFC300 column (5 µm, 7.8 mm ID × 15 cm) in isocratic PBS, pH 7.4 + 10% isopropanol. ~40 µg conjugate was injected for analysis.

UV-Vis analysis was performed on a NanodropOne spectrophotometer (Thermo Scientific, Waltham, MA, USA). The extinction coefficients in Table 1 were used to correct for Deruxtecan payload absorbance at 280 nm according to Equation (3).

(3) $${Corr.\;A}_{280}=A_{280}-A_{369}*A_{280}C.F.(DXd)$$

Equation (3). Corrected A_280_ absorbance for Trastuzmab–DXd conjugates

### 4.3. Trastuzumab NMR Sample Preparation

Lyophilized trastuzumab was solubilized in 1× PBS at a concentration of 20 mg/mL. 3 mL of trastuzumab was buffer exchanged with two passes through Thermo Scientific 7 kDa ZEBA columns (Waltham, MA, USA) equilibrated with 20 mM histidine, pH 5.5, according to the manufacturer’s protocol. The trastuzumab was then concentrated to 30.5 mg/mL with a 30 K MWCO Vivaspin centrifugal concentrator and filtered through a 0.22 µM sterile filter. The NMR sample for trastuzumab was prepared by dilution of the 30.5 mg/mL stock with D_2_O to generate a final sample of 29 mg/mL with 5% D_2_O in 20 mM histidine buffer, pH 5.5.

### 4.4. ADC NMR Sample Preparation

ADCs were stored in frozen aliquots at −80 °C at stock concentrations of 33.2 mg/mL (T-MMAE DAR2.3, ~151 kDa), 27.9 mg/mL (T-MMAE DAR4.3, ~154 kDa), 43.5 mg/mL (T-MMAE DAR7.9, ~158 kDa), 41.7 mg/mL (T-DXd DAR2.3, ~150 kDa), 34.9 mg/mL (T-DXd DAR4.2, ~152 kDa), and 35.9 mg/mL (T-DXd DAR8, ~156 kDa). Prior to NMR data collection, samples were thawed and small aliquots were diluted 1:10 for SEC reanalysis and 15:100, followed by further dilution as described below, for IC_50_ determination using a stock buffer of 20 mM histidine, pH 5.5. NMR samples were diluted using 20 mM histidine, pH 5.5, to generate sample concentrations between 23 and 27 mg/mL containing 5% D_2_O. Sample concentrations for NMR experiments were as follows: 27.7 mg/mL (T-MMAE DAR2.3), 23.2 mg/mL (T-MMAE DAR4.3), 27.2 mg/mL (T-MMAE DAR7.9), 27.8 mg/mL (T-DXd DAR2.3), 27 mg/mL (T-DXd DAR4.2), and 26.9 mg/mL (T-DXd DAR8). After NMR data collection, samples were removed from NMR tubes and diluted 1:10 for SEC reanalysis.

### 4.5. NMR Data Collection and Analysis

NMR data were collected at 700 MHz (^1^H) on a Bruker (Billerica, MA, USA) Avance NEO spectrometer running either TopSpin4.4.1 or TopSpin4.2.0. Data were collected using standard Bruker pulse programs, specifically the hos_afhmqcetgpph (HOS-XLAFHMQC) [39], which was specifically developed as a methyl-selective experiment for large proteins. We investigated multiple pulse sequences for data collection, focusing on SOFAST or ALSOFAST experiments to facilitate rapid data acquisition for low stability samples. While we saw the highest overall signal-to-noise in the AFHMQC (afhmqcgpphsf in the Bruker pulse program) [40,41], we saw superior signal-to-noise in well dispersed peaks using the HOS-XLAFHMQC and thus prioritized that experiment. For the HOS-XLAFHMQC, a relaxation delay of 0.5 s was used with a flip angle of 70° and shortened INEPT of 1.4 ms. Spectra were collected with 50 ms resolution in ^1^H using a spectral width of 13.7 ppm and 20 ms resolution in ^13^C with a spectral width of 30 ppm. Initial 32 scan experiments were collected to assess sample quality and were followed by longer (128 scan minimum) experiments to facilitate more detailed analysis. Data were collected at 37 °C unless otherwise noted, such as in Figure 1 and Supplementary Figures S1 and S2. For ADC samples, 32 scan experiments were collected sequentially overnight after collection of longer experiments to monitor sample degradation over time. 32 scan experiments were used for comparison of low DAR samples in Figure 2 and Figure 4, but the reduced signal in the high DAR samples necessitated the use of longer 128 scan experiments in Figure 3 and Figure 5 for best visual comparison of spectra. NMR data were processed using NMRPipe [42] and analyzed in NMRFAM-SPARKY [43]. 3D contour plots, as shown in the table of contents figure, were generated using COLMARvista [44].

### 4.6. Cell Line Expression and Growth

SK-BR3, SKOV-4, MCF-7, and PC-3 cell lines were acquired from ATCC. Cells were identified and certified free of contamination by the cell bank prior to shipping. Upon receipt, cells were stored in the vapor phase of liquid nitrogen. Once thawed, cells were expanded with their respective media according to ATCC guidelines and cell banks were established. Working stocks were allowed to grow to passage 3 before use in cell-based assays and were used at a maximum passage of 20.

### 4.7. IC_50_ Determination

On day 0 of the cytotoxicity assay, 2000 cells were plated on a 96-well tissue culture microplate. Cells were allowed to attach overnight in the incubator at 37 °C and 5% CO_2_. Test articles were diluted to 1 µM stocks using 1× PBS. On Day 1, 7-fold serial dilutions of the test articles were made using cell media as diluent. The dilution series was added to the plated cells in triplicate and incubated for 6 days at 37 °C, 5% CO_2_. On Day 6 of the Assay, CellTiter-glo reagent (Promega, Madison, WI, USA, Ref G7570) was used according to the manufacturer’s protocol to determine cell viability. The luminosity signal was normalized to untreated wells and plotted with Graphpad Prism 10.6.1 to obtain response curves.

## Figures and Tables

**Table 1:** Extinction coefficients of trastuzumab antibody and Deruxtecan.

ε_mAb_ 280 nm (1%)	14.81
ε_mAb_ 280 nm (M^−1^ cm^−1^)	219,188
ε_mAb_ 369 nm (M^−1^ cm^−1^)	0
ε_DXd_ 280 nm (M^−1^ cm^−1^)	6275
ε_DXd_ 369 nm (M^−1^ cm^−1^)	21,176
A280 C.F. (DXd)	0.296

## Data Availability

Data may be available upon request.

## Supplementary Materials

The following supporting information can be downloaded at: https://www.mdpi.com/article/10.3390/molecules30224490/s1, Table S1: Characterization of DAR and percent monomer before and after NMR data collection; Table S2: Characterization of cytotoxicity (IC_50_) of conjugated antibodies; Figure S1: Trastuzumab spectra improve with elevated temperature and experimental time; Figure S2: Specific spectroscopic signatures of trastuzumab remain even at low temperatures; Figure S3: Higher concentrations of trastuzumab improve signal-to-noise; Figure S4: Trastuzumab spectra are relatively stable over long periods of time; Figure S5: T-MMAE spectra show slight changes over time at low DAR; Figure S6: T-MMAE spectra show substantial changes over time at high DAR; Figure S7: T-MMAE displays target specific cytotoxicity across a range of DAR; Figure S8: T-DXd spectra show slight intensity changes with time at all DAR; Figure S9: Efficacy of T-DXd increases with DAR in SK-BR-3 cell line; Figure S10: LC-MS data of light chain and heavy chain of unconjugated trastuzumab; Figure S11: Schematic of T-MMAE conjugate and LC-MS of mc-vc-PAB-MMAE linker-payload; Figure S12: Schematic of T-DXd conjugate and LC-MS of mc-GFFG-DXd linker-payload; Figure S13: LC-MS data of light chain and heavy chain of T-MMAE conjugates; Figure S14: LC-MS data of light chain and heavy chain of T-DXd conjugates.

## Abbreviations

ADC	Antibody–drug conjugate
DAR	Drug-antibody ratio
NMR	Nuclear magnetic resonance
MMAE	monomethyl auristatin E
DXd	deruxtecan
T-MMAE	Trastuzumab-mc-vc-PAB-MMAE
T-DXd	Trastuzumab-deruxtecan
IC_50_	Half maximal inhibitory concentration
