# Supplementary material for: Assessment of ADC Higher Order Structure Through 2D NMR Analysis
Source: Molecules. 2025 Nov 20;30(22):4490. doi: 10.3390/molecules30224490 (PMC12655470; doi:10.3390/molecules30224490)
Supplement: Supplementary file 1 [file molecules-30-04490-s001.zip › molecules-3954921-supplementary.pdf]

Assessment of ADC Higher Order Structure Through 2D NMR Analysis

Emily M. Grasso [1,*], Angela N. Marquard [2], Zachary Sparta [1,2], David Fry [1] and Nareshkumar Jain [3]

[1]Platform Innovation, NJ Bio, Inc., Princeton, NJ, USA 08540; [2]Bioconjugation Research and Development, NJ Bio, Inc., Princeton, NJ, USA 08540; [3]Global Operations, NJ Bio, Inc., Princeton, NJ, USA 08540; *Correspondence: emily.grasso@njbio.com

Characterization of Trastuzumab Conjugates									
Sample		Pre NMR				Post NMR			
		DAR by:			% Monomer by:	DAR by:			% Monomer by:
Conjugate	Target DAR	LC-MS	RP-LC	HIC	SEC	LC-MS	RP-LC	HIC	SEC
T-MMAE	2	2.1	2.4	2.5	99.08	2.7	2.5	2.5	96.93
	4	4.1	4.4	4.3	98.75	4	4.4	3.9	89.75
	8	7.8	7.9	-	97.4	7.7	7.8	-	71.66
T-DXd	2	2.1	2.5	-	98.17	2.6	2.4	2.5	98.11
	4	3.9	4.4	-	97.78	4.2	4.2	-	96.62
	8	8	7.9	-	98.39	8	8	-	97.26

Supplementary Table S1. Characterization of DAR and percent monomer before and after NMR data collection

Percent monomer from SEC and DAR for each conjugated species as calculated by equations 1 and 2 in Materials and Methods section 4.2 using samples from before and after NMR data collection.

IC50 Assay Results					
Sample		IC50 (pM)			
		Cell Line			
Conjugate	DAR	SK-BR-3	SK-OV-3	MCF-7	PC-3
T-MMAE	2.3	22.3	196	-	-
	4.3	19.6	148	230000	-
	7.9	15.4	67.2	1140	240000
T-DXd	2.3	243	-	-	-
	4.2	129	-	-	-
	8	65.7	-	-	-

Supplementary Table S2. Characterization of cytotoxicity (IC_{50}) of conjugated antibodies

IC_{50} values from cytotoxicity assays with DAR series for T-MMAE and T-DXd shown in Supp. Fig. 7 and 9 reported in pM.

Supplementary Figures:

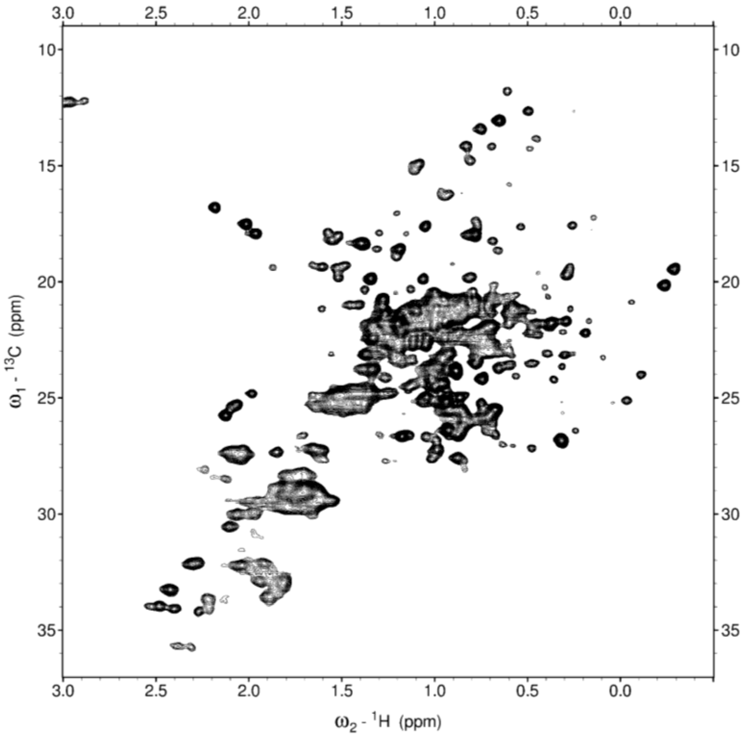

Supplementary Figure S1. Trastuzumab spectra improve with elevated temperature and experimental time

{^{1}H-^{13}C}HOS-XLAFHMQC of 29 mg/mL trastuzumab in 20 mM histidine, pH 5.5, at 50°C with 128 scans shows improved signal-to-noise over spectra collected with fewer scans or lower temperature though peaks largely remain in the same location.

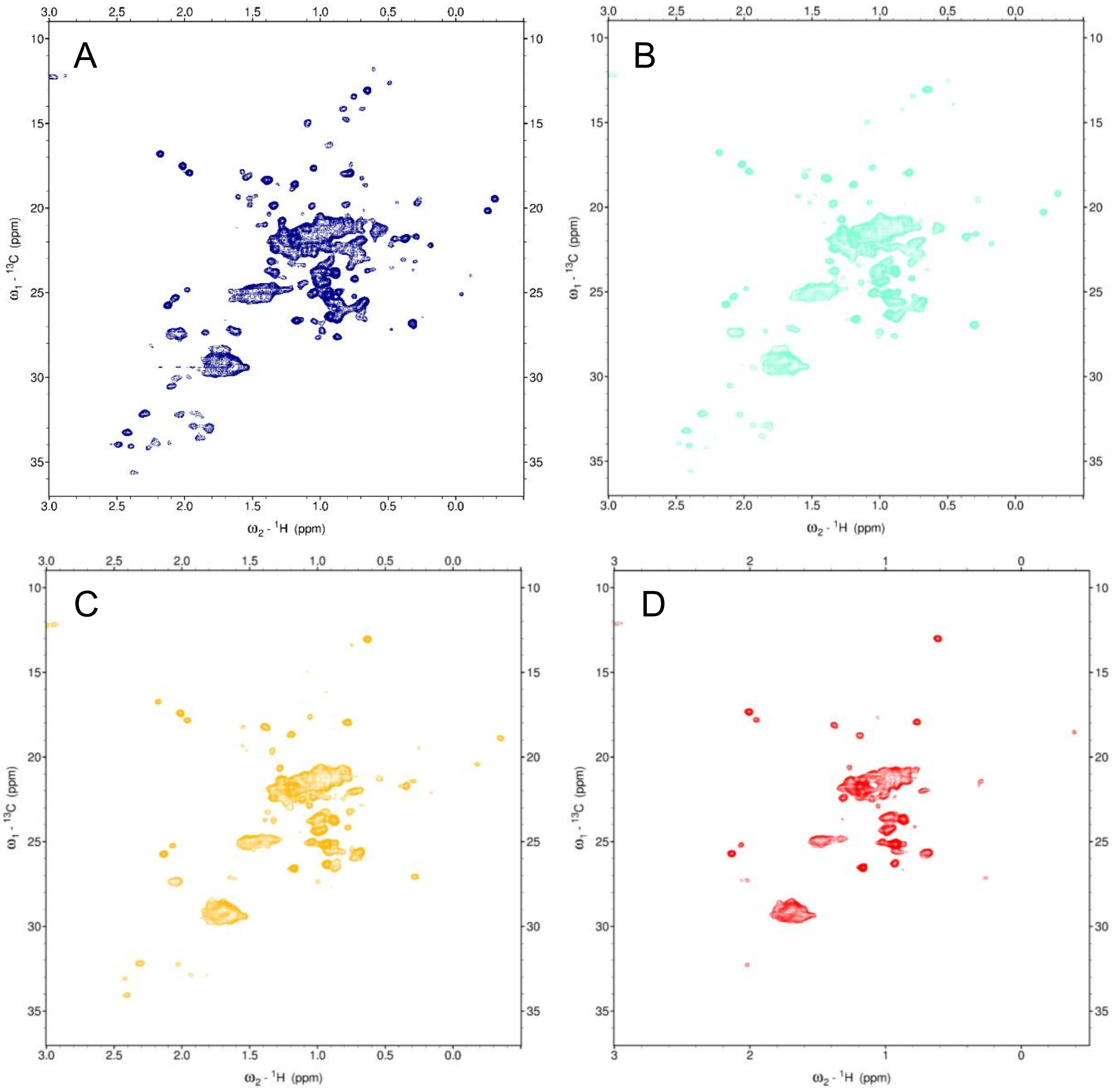

Supplementary Figure S2. Specific spectroscopic signatures of trastuzumab remain even at low temperatures

{^{1}H-^{13}C}HOS-XLAFHMQC of 29mg/mL trastuzumab in 20 mM histidine, pH 5.5, with 32 scans at (A) 50°C, (B) 40°C, (C) 30°C, (D) 20°C show improved signal at high temperatures, but specific peaks linger even at the lowest temperatures.

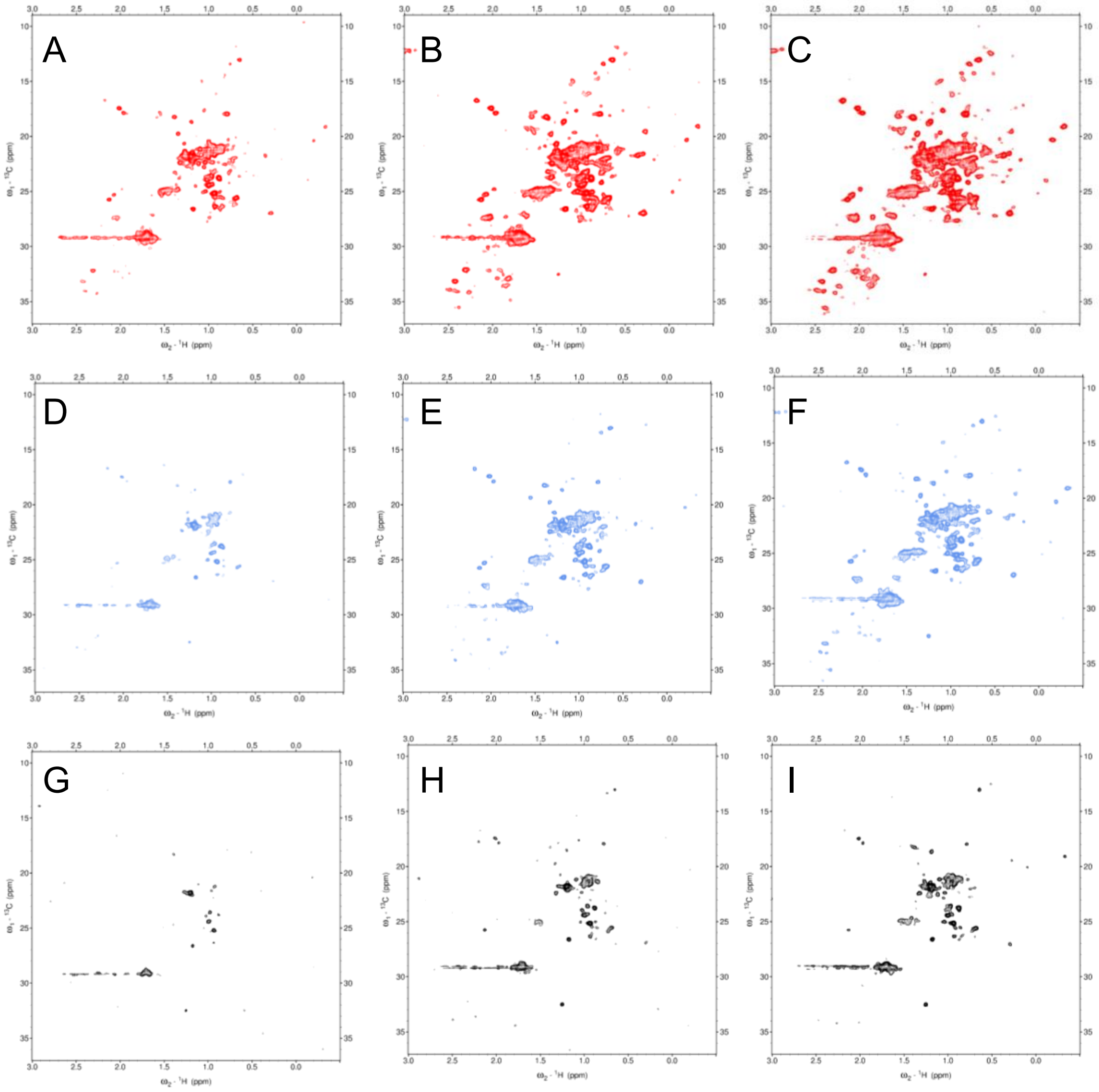

Supplementary Figure S3. Higher concentrations of trastuzumab improve signal-to-noise

$\{^{1}H$-$^{13}C\}$HOS-XLAFHMQC at 37°C of (A-C) 10 mg/mL trastuzumab with (A) 32, (B) 128 and (C) 256 scans; (D-F) 5 mg/mL trastuzumab with (D) 32, (E) 128 and (F) 256 scans; and (G-I) 2.5 mg/mL trastuzumab with (G) 32, (H)128 and (I) 256 scans. With sufficient scans, many well-dispersed peaks can be seen at 10 mg/mL trastuzumab, but those peaks are not visible at lower concentrations.

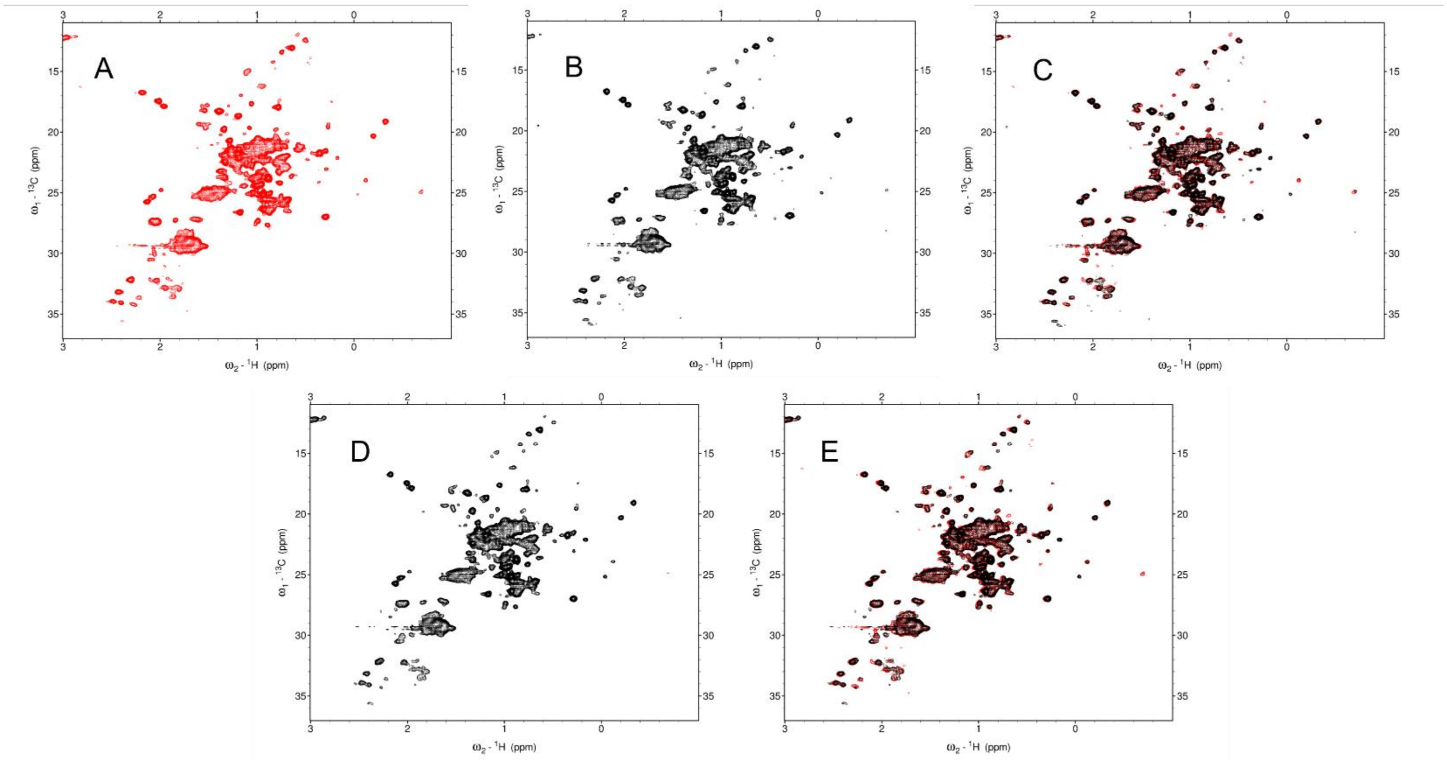

Supplementary Figure S4. Trastuzumab spectra are relatively stable over long periods of time

(A) Initial {^{1}H-^{13}C}HOS-XLAFHMQC at 37°C of 29 mg/mL trastuzumab sample collected with 32 scans and (B) {^{1}H-^{13}C}HOS-XLAFHMQC at 37°C of 29 mg/mL trastuzumab collected after approximately one day show minimal changes, as is evident from the overlaid spectra in (C). The stability of this sample is further corroborated by (D) the same spectrum collected after over 4 months on the same sample after multiple rounds of data collection and prolonged storage at 4°C, as is evident from the overlaid spectra in (E).

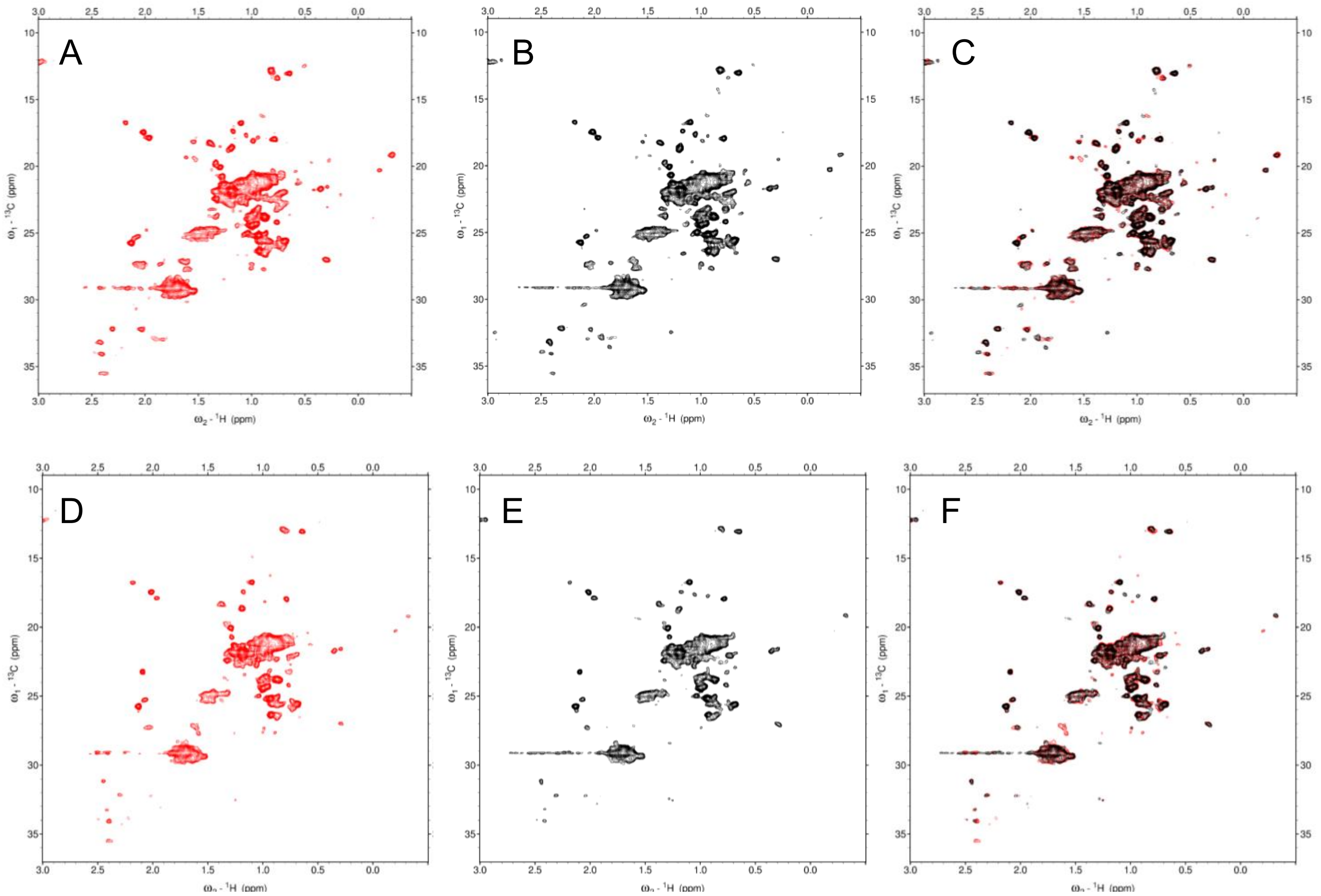

Supplementary Figure S5. T-MMAE spectra show slight changes over time at low DAR.

(A) Initial spectrum of DAR2.3 (red), (B) final spectrum of DAR2.3 (black), and (C) overlay of (A) and (B) indicate that the spectra for this sample changed slightly overthe course of approximately one day. (D) Initial spectrum of DAR4.3 (red), (E) final spectrum of DAR4.3 (black), and (F) overlay of (D) and (E) indicate that the spectra for this sample changed slightly over time. All spectra were collected with 32 scans.

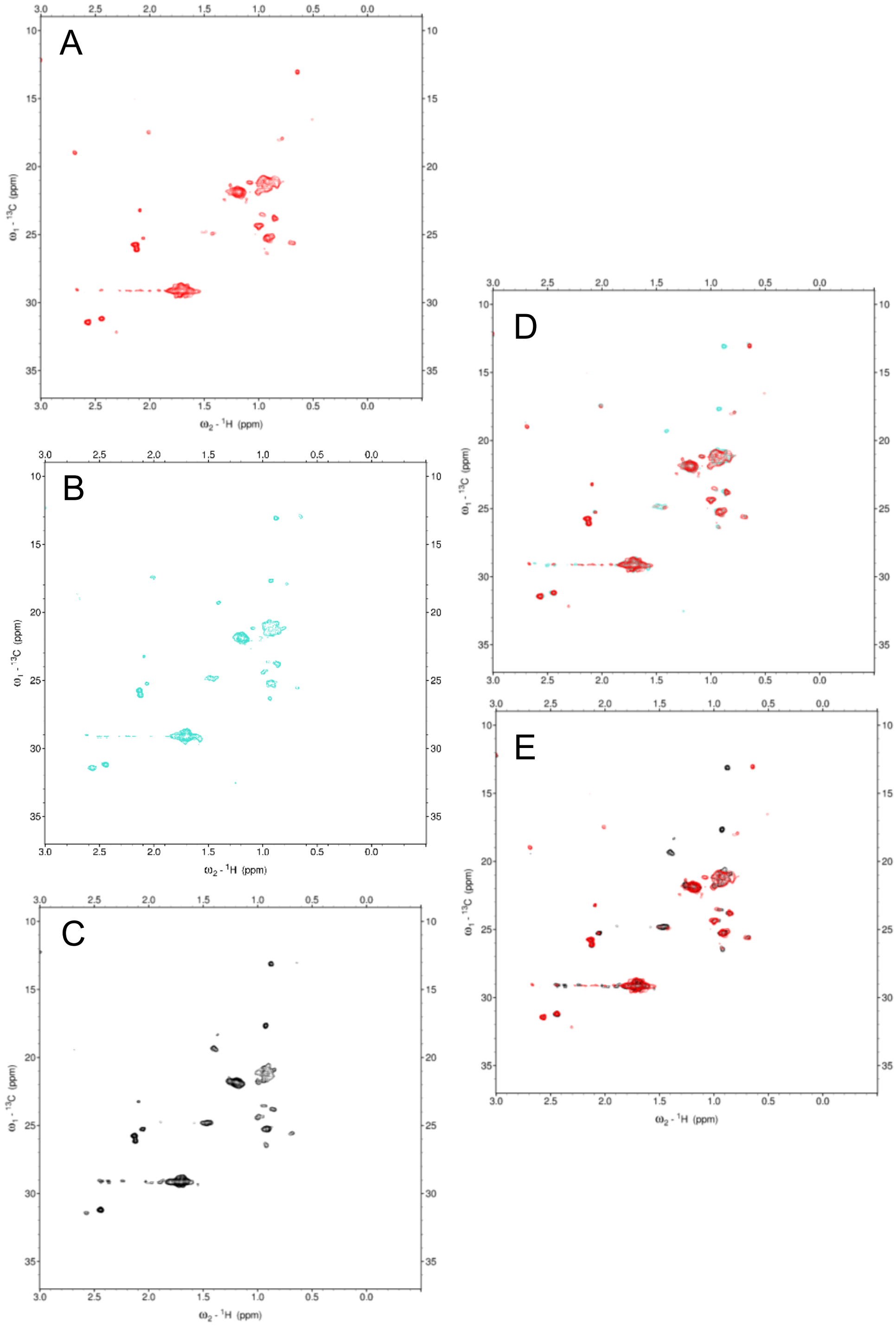

Supplementary Figure S6. T-MMAE spectra show substantial changes over time at high DAR. The NMR spectra of T-MMAE DAR7.9 show substantial changes over time going from (A) the initial spectrum collected to (B) an intermediate spectrum collected after approximately 7.5 hours to (C) a final spectrum collected after approximately 20 hours. This is apparent in (D) overlaid spectra from (A) and (B) and (E) overlaid spectra from (B) and (C). All spectra were collected with 32 scans.

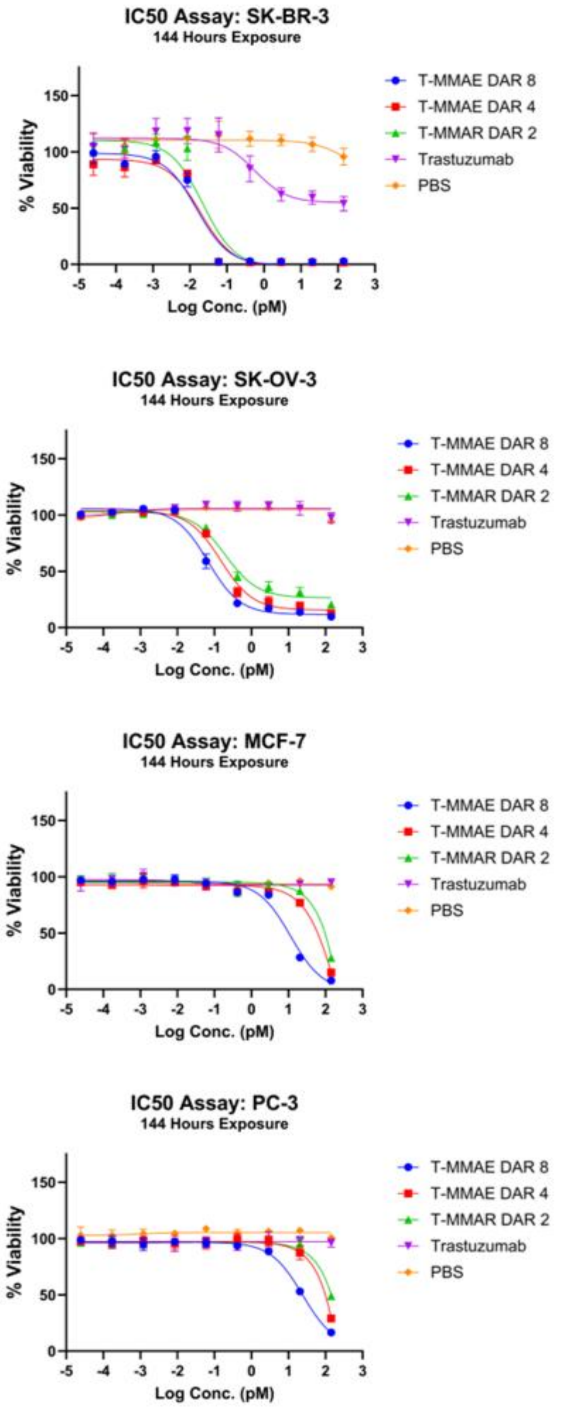

Supplementary Figure S7. T-MMAE displays target specific cytotoxicity across a range of DAR

High HER2 expressing cell lines SK-BR-3 and SK-OV-3 show similar responses to the DAR series of T-MMAE, with SK-BR-3 being more sensitive to the treatments. Low HER2 expressing cell lines MCF-7 and PC-3 were impacted at higher concentrations by the DAR 8 T-MMAE more significantly than the DAR 2 and DAR 4 treatments.

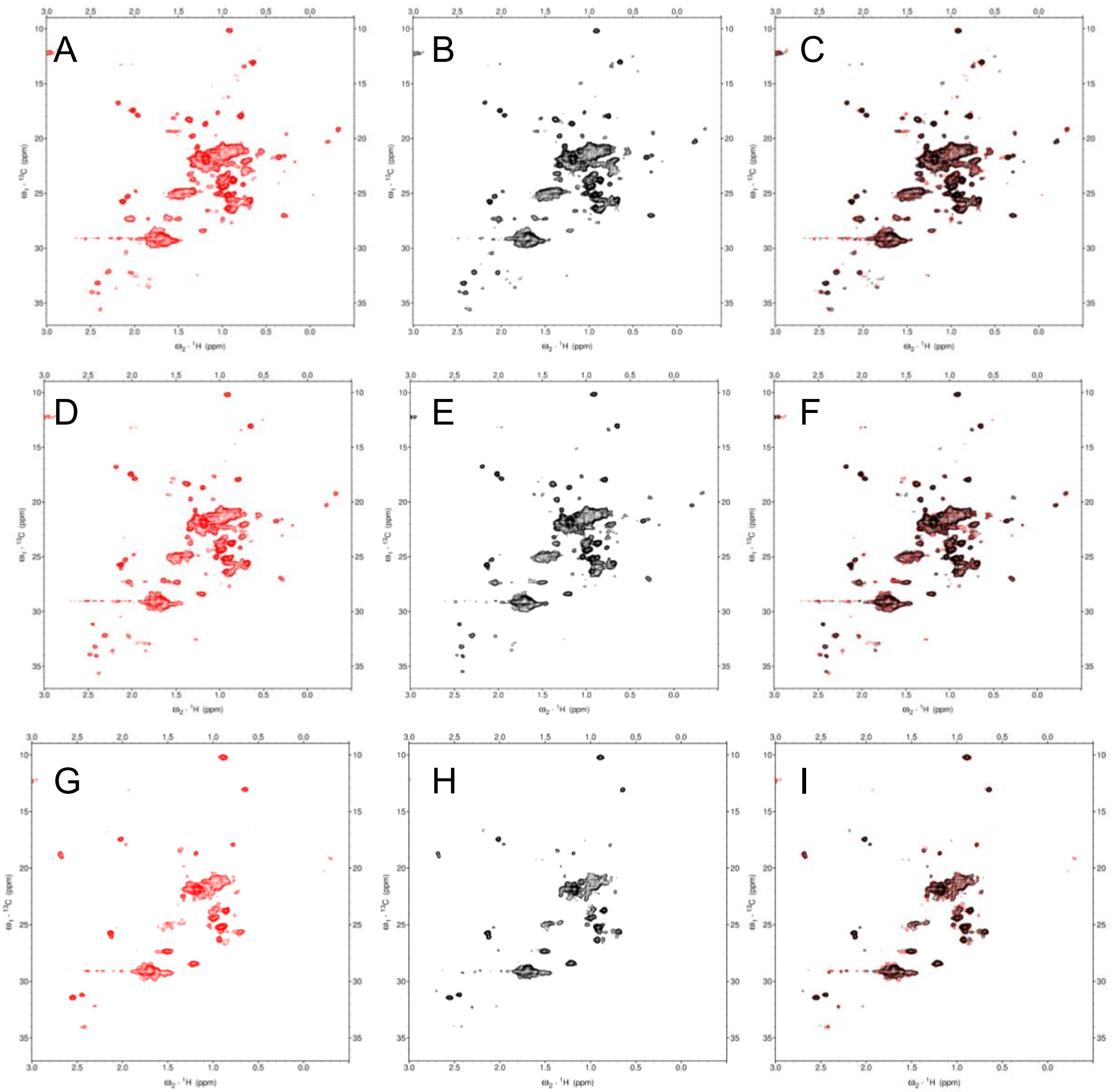

Supplementary Figure S8. T-DXd spectra show slight intensity changes with time at all DAR

(A) Initial spectrum of T-DXd DAR2.3, (B) final spectrum of T-DXd DAR2.3 and (C) overlay of (A) and (B) reveal minimal intensity changes as a function of time. Similarly (D) initial spectrum of T-DXd DAR4.2, (E) final spectrum of T-DXd DAR4.2 and (F) overlay of (D) and (E) show slight changes over time, as do (G) initial spectrum of T-DXd DAR8, (H) final spectrum of T-DXd DAR8 and (I) overlaid (G) and (H). All spectra were collected with 32 scans over the course of approximately one day.

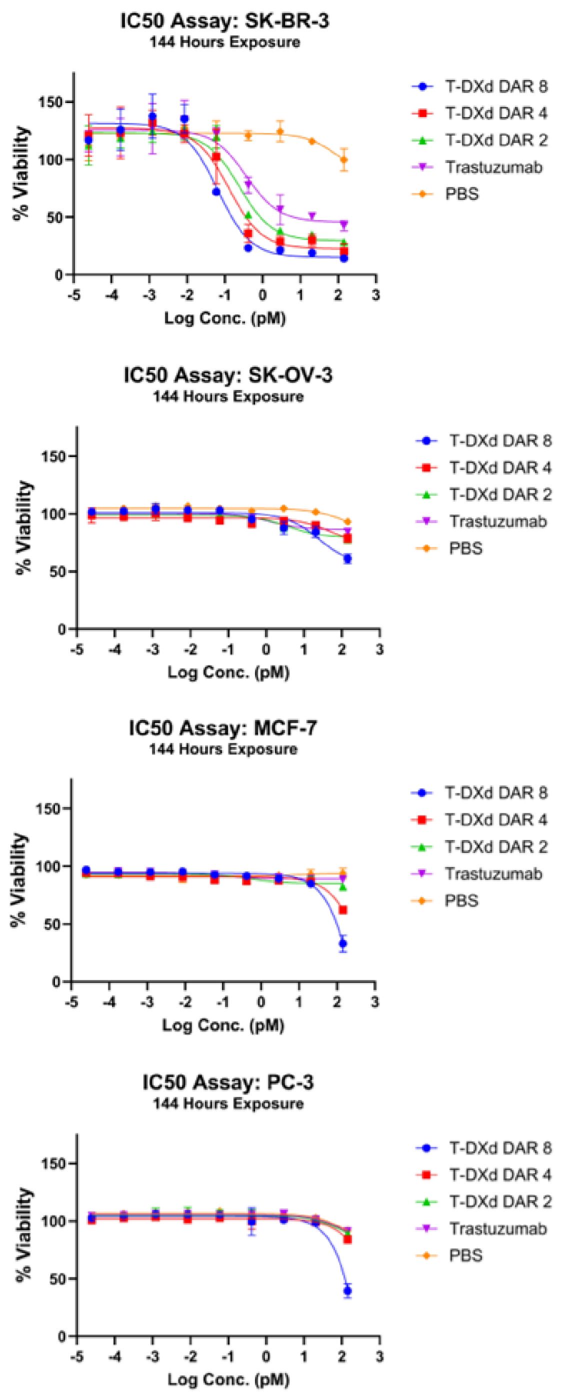

Supplementary Figure S9. Efficacy of T-DXd increases with DAR in SK-BR-3 cell line

Cytotoxicity assays with DAR series T-DXd showed a response in the high HER2 expressing cell line SK-BR-3 and no response from SK-OV-3 or the low HER2 expressing lines MCF-7 and PC-3.

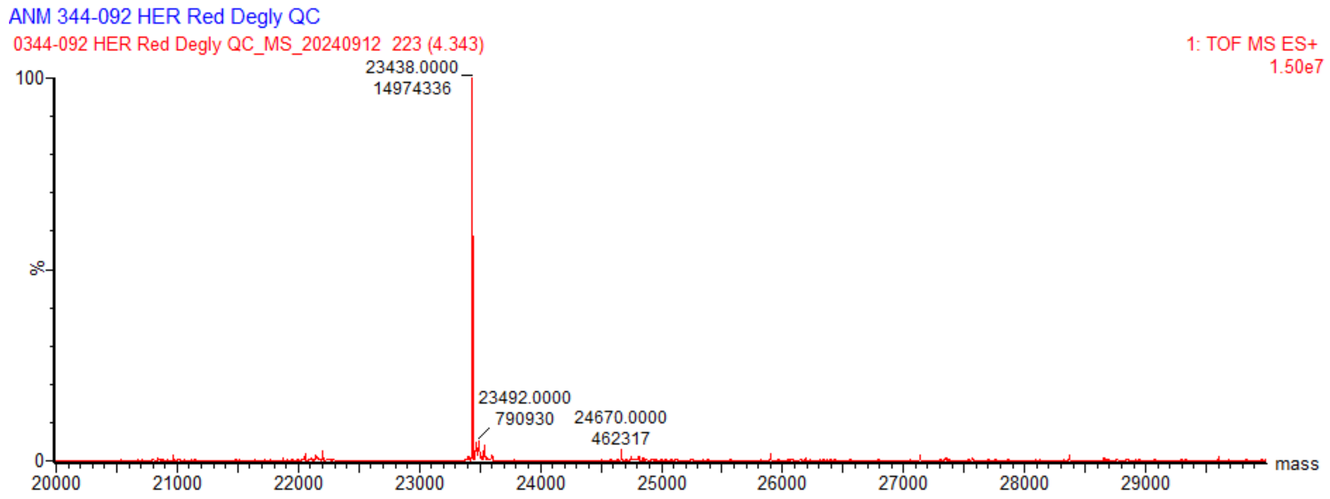

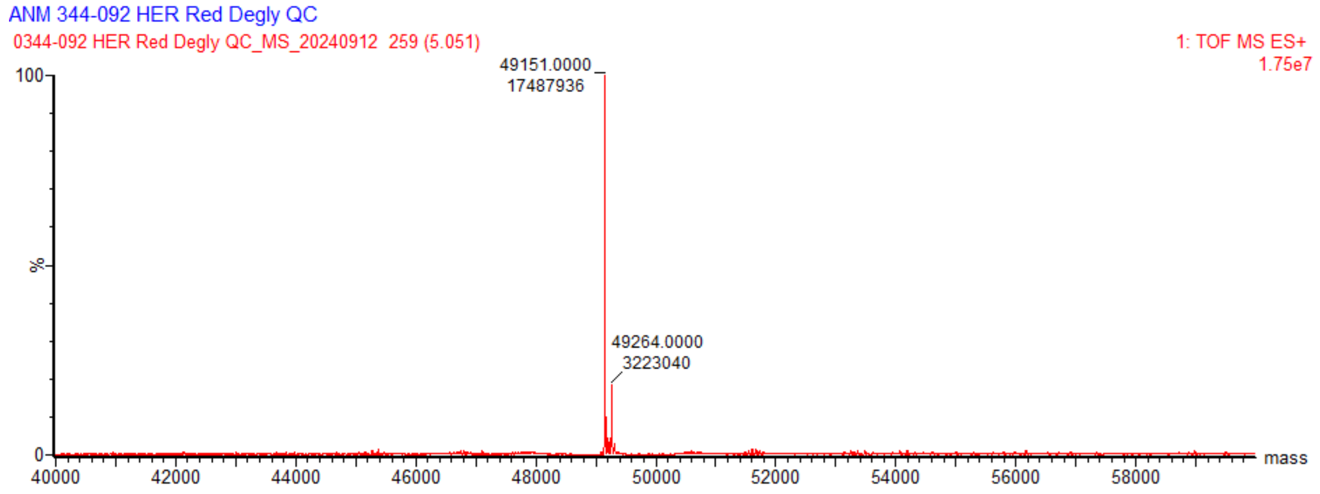

Supplementary Figure S10. LC-MS data of light chain and heavy chain of unconjugated trastuzumab

LC-MS data for both light and heavy chains of deglycosylated trastuzumab prior to conjugation are consistent with expected masses for the antibody

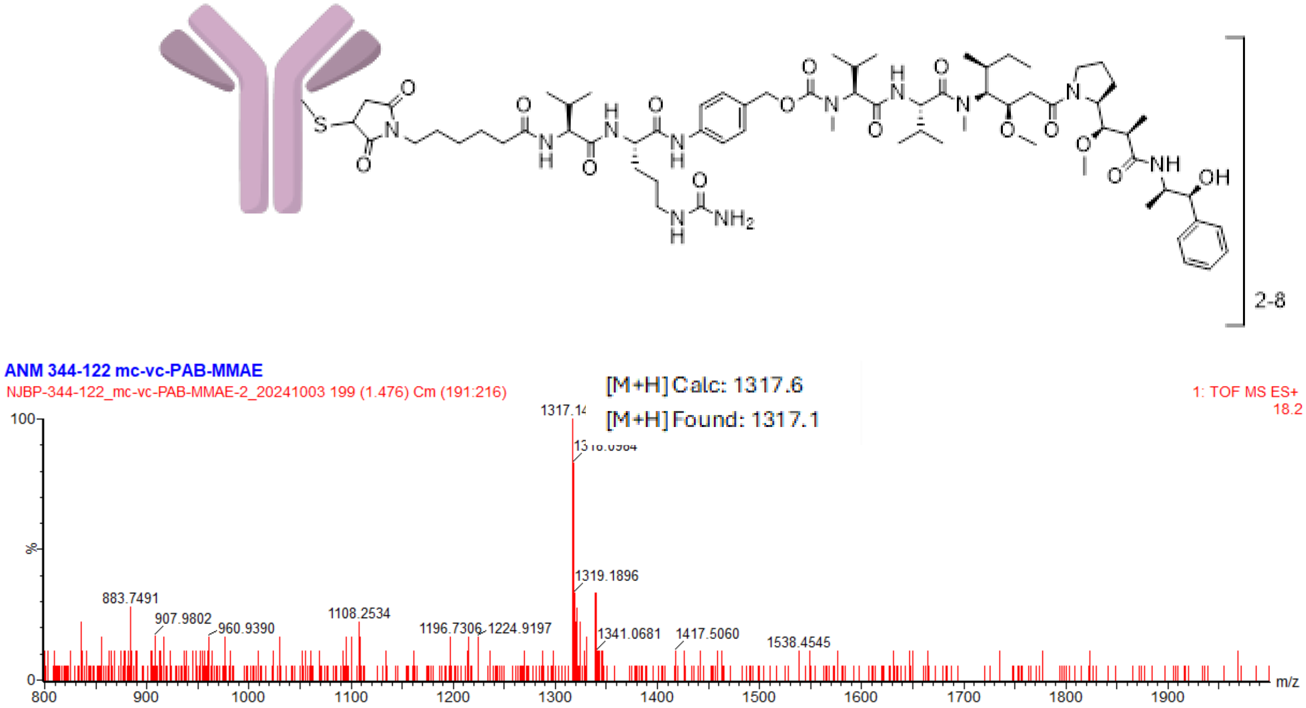

Supplementary Figure S11. Schematic of T-MMAE conjugate and LC-MS of mc-vc-PAB-MMAE linker-payload

Schematic of T-MMAE after conjugation along with LC-MS data of the linker-payload prior to conjugation.

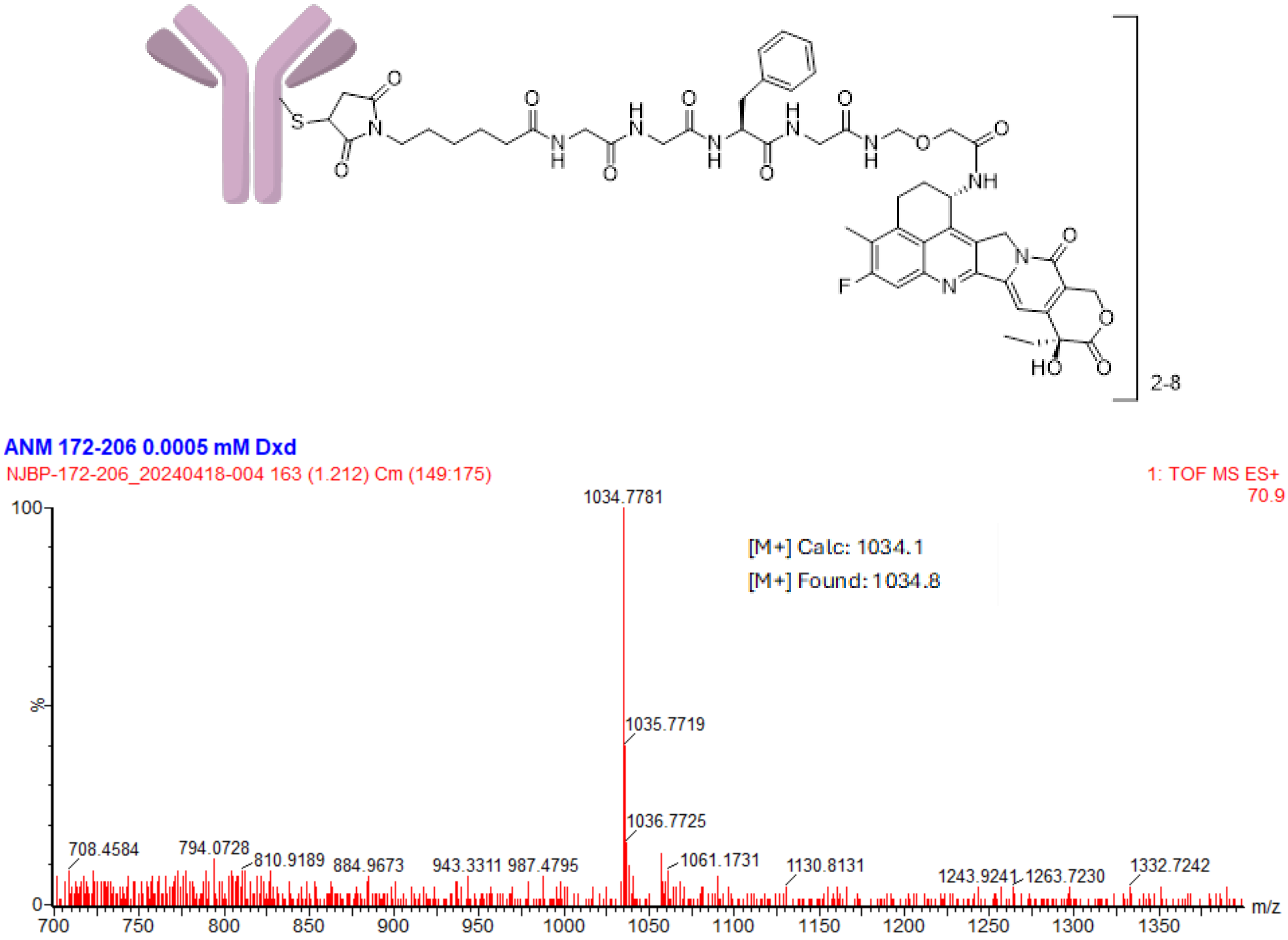

Supplementary Figure S12. Schematic of T-DXd conjugate and LC-MS of mc-GFFG-DXd linker-payload

Schematic of T-DXd after conjugation along with LC-MS data of the linker-payload prior to conjugation.

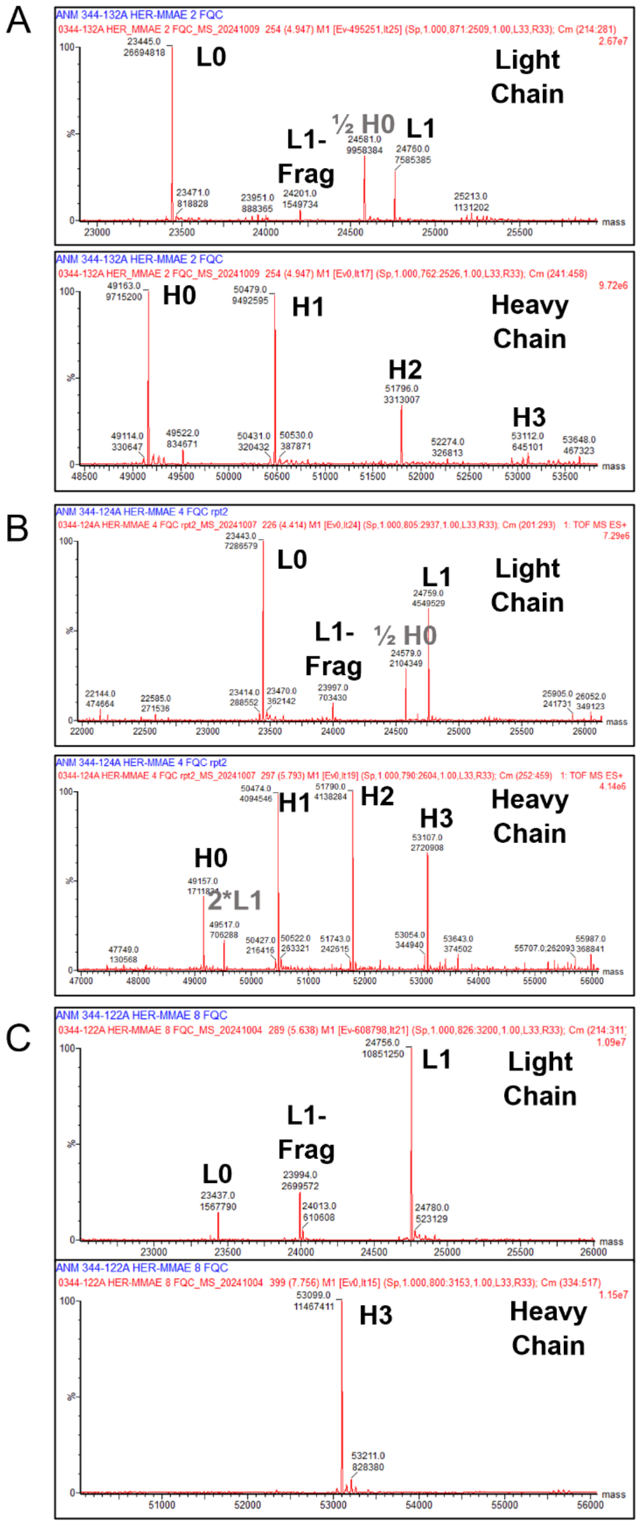

Supplementary Figure S13. LC-MS data of light chain and heavy chain of T-MMAE conjugates

LC-MS data for (A) T-MMAE DAR2, (B) T-MMAE DAR4, and (C) T-MMAE DAR8 show conjugations to both light and heavy chain which were used for subsequent DAR calculations

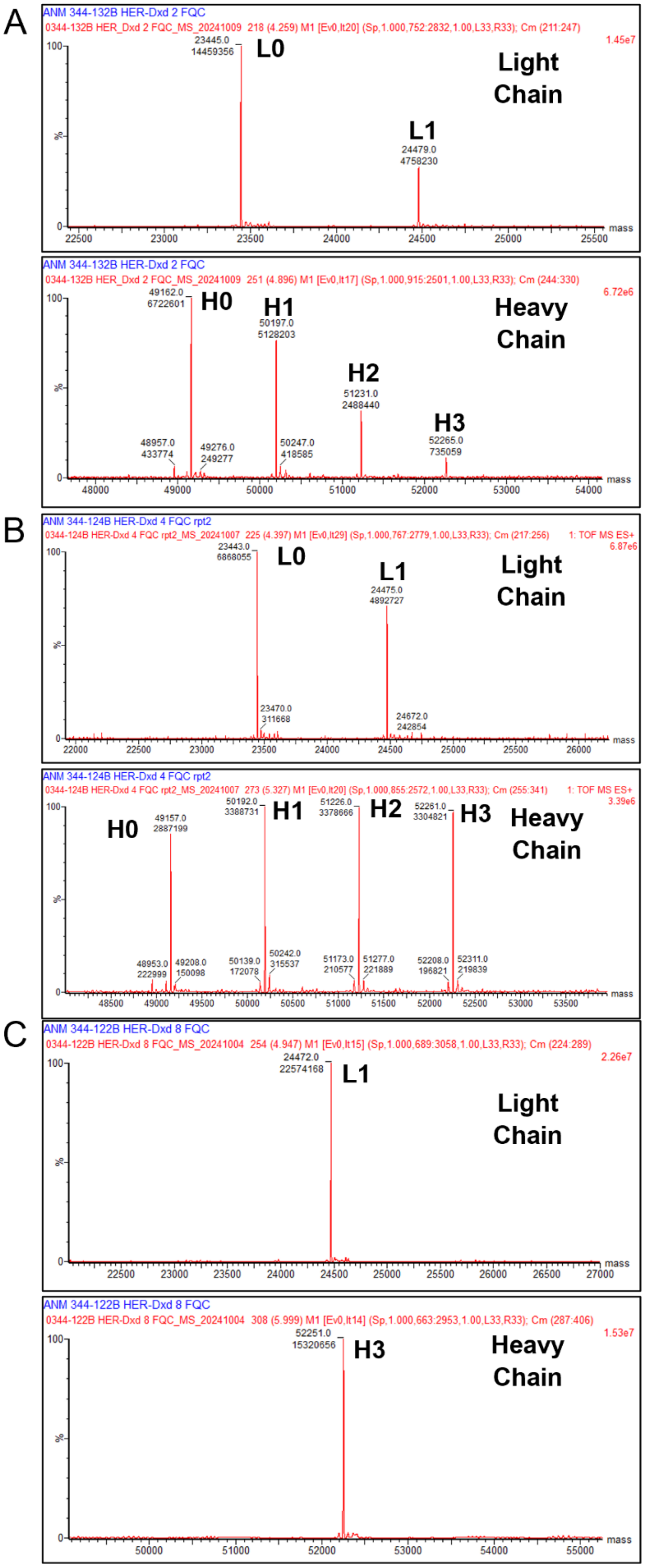

Supplementary Figure S14. LC-MS data of light chain and heavy chain of T-DXd conjugates

LC-MS data for (A) T-DXd DAR2, (B) T-DXd DAR4, and (C) T-DXd DAR8 show conjugations to both light and heavy chain which were used for subsequent DAR calculations.
